# Synthetic Biology and Biosecurity: Challenging the “Myths”

**DOI:** 10.3389/fpubh.2014.00115

**Published:** 2014-08-21

**Authors:** Catherine Jefferson, Filippa Lentzos, Claire Marris

**Affiliations:** ^1^Department of Social Science, Health and Medicine, King’s College London, London, UK

**Keywords:** synthetic biology, biosecurity, bioterrorism, biological weapons, DIY biology, iGEM, policy discourse, non-proliferation

## Abstract

Synthetic biology, a field that aims to “make biology easier to engineer,” is routinely described as leading to an increase in the “dual-use” threat, i.e., the potential for the same scientific research to be “used” for peaceful purposes or “misused” for warfare or terrorism. Fears have been expressed that the “de-skilling” of biology, combined with online access to the genomic DNA sequences of pathogenic organisms and the reduction in price for DNA synthesis, will make biology increasingly accessible to people operating outside well-equipped professional research laboratories, including people with malevolent intentions. The emergence of do-it-yourself (DIY) biology communities and of the student iGEM competition has come to epitomize this supposed trend toward greater ease of access and the associated potential threat from rogue actors. In this article, we identify five “myths” that permeate discussions about synthetic biology and biosecurity, and argue that they embody misleading assumptions about both synthetic biology and bioterrorism. We demonstrate how these myths are challenged by more realistic understandings of the scientific research currently being conducted in both professional and DIY laboratories, and by an analysis of historical cases of bioterrorism. We show that the importance of tacit knowledge is commonly overlooked in the dominant narrative: the focus is on access to biological materials and digital information, rather than on human practices and institutional dimensions. As a result, public discourse on synthetic biology and biosecurity tends to portray speculative scenarios about the future as realities in the present or the near future, when this is not warranted. We suggest that these “myths” play an important role in defining synthetic biology as a “promissory” field of research and as an “emerging technology” in need of governance.

## Introduction

“Synthetic biology strives to make the engineering of biology easier and more predictable.” [(1), p. 6]

A dominant narrative has emerged in policy arenas, in which advances in the biosciences are seen to make biology easier and more accessible, and this is presumed to increase the so-called “dual-use” threat, i.e., the potential for the same scientific research to be “used” for peaceful purposes or “misused” for warfare or terrorism. Developments in synthetic biology, a field that emerged at the start of the twenty-first century with the stated aim of “making biology easier to engineer” (1, 2), have further fueled these concerns. Fears have been expressed that synthetic biology will lead to further “de-skilling” and that, combined with open online access to the genomic DNA sequences of pathogenic organisms and the reduction in price for DNA synthesis, this will make biology increasingly accessible to people operating outside well-equipped professional research laboratories, including people with malevolent intentions. The emergence of do-it-yourself (DIY) biology communities and the student iGEM competition has come to epitomize this supposed trend toward greater ease of access and the associated potential threat from rogue actors.

In this article, we analyze this dominant narrative and identify five “myths” that permeate discussions about synthetic biology and biosecurity. We describe each of these myths and provide illustrative examples of how they are deployed in policy arenas. We then demonstrate how each of these myths is challenged by more realistic understandings of the scientific research currently being conducted in both professional and DIY laboratories, and by an analysis of historical cases of bioterrorism. In particular, we show that the importance of tacit knowledge is commonly overlooked in the dominant narrative: the focus is on access to biological materials and digital information, rather than on human practices and institutional dimensions. Sonia Ben Ouagrham-Gormley and Kathleen Vogel have argued, on the basis on their in-depth analysis of the US and Soviet biowarfare programs, that there are important intangible barriers to the proliferation of biological weapons (3–5). These authors show how tacit knowledge has been marginalized in assessments of the dual-use threat of biotechnologies in the twenty-first century.

Tacit knowledge is crucial to conduct advanced bioscience research, and is by definition difficult to share. This is encapsulated by Polanyi’s remark that “*we can know more than we can tell*” [(6), p. 4, emphasis in original]. As a result, researchers who work within institutionalized laboratories acquire tacit knowledge through experience, by working in teams and participating in professional scientific networks. But acquiring tacit knowledge is much more difficult for people who operate outside of such institutions, such as DIY biologists and bioterrorists. Broadly, tacit knowledge refers to skills and techniques that cannot be readily codified but, rather, are acquired through a process of “learning by doing” or “learning by example,” and often take considerable time and effort to gain. According to Harry Collins, a distinction can be made between “weak,” “somatic limit,” and “collective” tacit knowledge (7, 8). Revill and Jefferson (9) have drawn on Collins’ classification to explore the importance of tacit knowledge in the practice of synthetic biology and the conduct of bioweapons programs. They explain that “[w]eak tacit knowledge is that which could, under certain circumstances, be rendered explicit but either through inability, unwillingness, or practicality remains unwritten and implicit” [(9), p. 3]. Individual, or somatic, tacit knowledge “refers to things that our bodies can do, which we cannot articulate, transfer and replicate as knowledge without the recipient learning by doing” (*ibid*., p. 4–5). These are the skills, mechanical techniques, and idiosyncratic know-how obtained by individuals through trial-and-error problem-solving or through a master-apprentice style relationship. Collective, or communal, tacit knowledge “is the combined knowledge that is developed through interaction between experts with different disciplinary backgrounds working together” (*ibid*., p. 6). This can be conceptualized “as the bringing together of different disciplinary experts that are greater than the sum of their parts” (*ibid*., p. 6–7), or, following Vogel (10), can be understood as “communally synthesized tacit knowledge” that comes from the ongoing interactions between different types of expertise. Revill and Jefferson (9) provide examples of tacit knowledge from each of these categories from the history of biological weapons programs and the practice of advanced biological sciences, including synthetic biology.

Ben Ouagrham-Gormley and Vogel, and Revill and Jefferson, argue that a better understanding of tacit knowledge could improve the assessments of the dual-use threat posed by modern biotechnologies. Yet, tacit knowledge continues to be overlooked in policy arenas. In this paper, we examine the way in which the biosecurity threat posed by synthetic biology has been framed within the dominant narrative that permeates scientific and policy arenas. We identify five recurring “myths” that emerge from this analysis:

- Myth 1: synthetic biology is de-skilling biology and making it easier for terrorists to exploit advances in the biosciences;
- Myth 2: synthetic biology has led to the growth of a DIY biology community, which could offer dual-use knowledge, tools, and equipment for bioterrorists seeking to do harm;
- Myth 3: DNA synthesis has become cheaper and can be out-sourced, and this will make it easier for terrorists to create biological threat agents;
- Myth 4: synthetic biology could be used to design radically new pathogens;
- Myth 5: terrorists want to pursue biological weapons for high consequence, mass casualty attacks.

The use of the term “myths” is not intended here to imply falsity. We are not simplistically opposing “myth” and “reality,” and we are not arguing that there is no threat. Rather, our aim is to convey the pervasiveness of misleading assumptions about both synthetic biology and bioterrorism that frequently underlie discussions about the dual-use threat of synthetic biology, and to draw out some of the subtleties that frequently disappear from these discussions. Moreover, we do acknowledge that these myths have power and perform real functions such as mobilizing support, resources, and action. Thus, the dominant narrative identified in this paper helps to bring into being a particular hoped-for future, and attributes roles and influence to different actors. It influences the way in which the problem is defined, and thus the kinds of solutions that are proposed. These “myths” are real enough to influence policy in significant ways and that why it is important to examine them more carefully.

## Materials and Methods

The research presented here draws on participant observation in scientific and policy arenas, and on a review of a wide range of written materials.

All three authors have been participant-observers in either synthetic biology arenas, or biosecurity arenas, or both, for a number of years. Filippa Lentzos has been regularly attending and actively participating in a wide range of events on biosecurity, biological arms control and non-proliferation for over a decade. Catherine Jefferson has been involved in discussions on bioweapons, biosecurity and arms control for a decade. Claire Marris has been attending and participating in a wide range of scientific and policy events on synthetic biology for 5 years. Filippa Lentzos has been engaged in the field of synthetic biology for the last 7 years. The synthetic biology events include scientific meetings ranging from large-scale international conferences such as those in the SBx.0 series to laboratory meetings at the Centre for Synthetic Biology and Innovation (CSynBI) that all three authors are members of, or informal conversations with CSynBI and other collaborators in the field of synthetic biology. Our involvement also includes participation in expert committees and working groups, and public debates organized by scientific organizations.^1^

The key insights reported in this paper emerged from this immersion in the worlds of synthetic biology and biosecurity, which provided the authors with regular opportunities to interact with synthetic biologists, government officials, security analysts, technical experts, diplomats, public health officials, law enforcement agents, DIY biologists, and others who have assembled around the “problem” of synthetic biology “misuse.” These interactions took place in “natural” settings (as opposed to, for example, an interview setting), in places and during events that these actors – and the authors – were participating in through the course of their work.

It is through this fieldwork that we became aware of the prevalence of particular ways of framing the issues at stake, and were able to analyze how actors mobilized particular arguments. This was complemented by a review of written materials, which has been utilized mostly to confirm the hypotheses developed through our fieldwork, and to select citations to illustrate our results. This was necessary because many of the meetings that we participated in were not public and/or were not recorded, so it is not technically possible to provide verbatim quotations from those events. Moreover, in most cases, providing such quotations would not be compatible with research ethics. The documents reviewed are mostly from the “gray” literature: reports produced by scientific and biosecurity institutions. But they also include relevant academic articles, websites, blogs, and print media. The key criteria for selection of documents and citations were that they should be produced or written by key institutions or influential individuals in the fields of synthetic biology and/or biosecurity, for example: Drew Endy, Rob Carlson, George Church as leaders in the field of synthetic biology; Jonathan Tucker, Tara O’Toole, and Laurie Garrett as US experts in the field of biosecurity; Markus Schmidt as a key European commentator on “ethical, legal, and social issues” related to synthetic biology; US government officials and politicians; and institutions such as the Biological Weapons Convention (BWC), the European Commission, the US National Academy of Science, the US National Research Council, the US National Advisory Board for Biosecurity (NSABB), the J. Craig Venter Institute (JCVI) and the UK Royal Academy of Engineering. Moreover, the illustrative citations are taken mostly from documents and from (individual or institutional) authors that are themselves routinely cited by actors in discussions about synthetic biology and biosecurity.

Ethnographic data from participant observation and the literature review was complemented by a 1-day workshop convened by the authors at King’s College London on 28th February 2014 (11). This workshop brought together a group of 23 scientists, policy experts, science journalists, and social scientists (mostly from the UK) with specialist expertise in either synthetic biology or biosecurity (or both). A draft of the present paper was circulated in advance of the workshop and participants were asked to comment on it. The comments received and the discussions that occurred during the workshop provided additional information and confirmation of our hypotheses.

## Results

### Myth 1

#### Synthetic biology is de-skilling biology and making it easier for terrorists to exploit advances in the biosciences

Founding leaders in synthetic biology have argued that developments in the field would lead to a situation where biology would not only become “easier to engineer,” but that it would become easier for *anyone* to engineer biology. For example, during his early campaigns to garner political and financial support for the field, Drew Endy stressed that synthetic biology would lead to “the probable inability to control the distribution of technologies needed to manipulate biological systems” (12). Rob Carlson, in an article published in a biosecurity journal, emphasized that it would lead to the “inevitable” “proliferation of skills” [(13), p. 7]. Endy and Carlson both pointed to potential dual-use threats, whereby the powerful technology that they were promoting could be misapplied for harmful purposes. George Church also raised these issues in his “Synthetic Biohazard Non-proliferation Proposal” (14). The JCVI funded a report on “Options for Governance” that also focused almost exclusively on such risks (15).

The idea that synthetic biology could make it easier for non-specialists, including those working outside of institutions, to exploit this powerful technology for both benevolent and malevolent purposes, has to a large extent become a hallmark of the field. For example, in an article entitled “Diffusion of synthetic biology: a challenge to biosafety” Markus Schmidt, who was the leader of the first European Commission-funded project on the “Ethical, Legal, and Social Issues” of the field (SYNBIOSAFE) and who has become a prominent commentator on the risks involved, has argued, in a paper that has been cited 52 times in Google Scholar (accessed 10th July 2014), that:

With this “de-skilling” agenda, synthetic biology might finally unleash the full potential of biotechnology and spark a wave of innovation, as more and more people have the necessary skills to engineer biology [(16), p. 1].

This portrayal of synthetic biology focuses on the powerful positive impact that could be “unleashed” by “de-skilling,” and inevitably leads to concerns that such power could fall into the hands of people with malevolent intentions. As a result, policy experts have routinely expressed concerns that synthetic biology could be used by terrorists to produce biological weapons. For example, political scientists from the Massachusetts Institute of Technology (Gautam Mukunda^2^ and Kenneth Oye), who were both at the time working for the US Synthetic Biology Research Center (Synberc), published an article on synthetic biology and biosecurity in 2009, in which they stated:

Synthetic biology includes, as a principal part of its agenda, a sustained, well-funded assault on the necessity of tacit knowledge in bioengineering and thus on one of the most important current barriers to the production of biological weapons [(17), p. 14].

The European Group on Ethics in Science and New Technologies to the European Commission also emphasized this in their 2009 Opinion on synthetic biology:

Ethical issues arise particularly from dangers of using synthetic lethal and virulent pathogens for terrorist attacks, bio-war, or maleficent uses (“garage terrorism”, “bio-hacking”), particularly if knowledge and skills on how to produce such pathogens are freely available [(18), p. 43].

#### Challenges to Myth 1

These concerns are based on the assumption that synthetic biology already has made it, or shortly will make it, easy *for anybody* to “engineer biology.” The underlying vision is one where well-characterized biological “parts” can be easily obtained from open-source online registries and then easily assembled, by people with no specialist training and working outside professional scientific institutions, into genetic “circuits,” “devices,” and “systems” that will reliably perform desired functions in live organisms (1, 2). However, this does not even reflect current realities in academic or commercial science laboratories, where researchers are still struggling with every stage of this process (19, 20).

Moreover, synthetic biologists who participated in our recent workshop (11) argued that although historical experience with other forms of (non-biological) engineering demonstrate that dependence on the craft skills of a small number of highly trained individuals is reduced for some parts of the production process, usually by standardization and mechanization, this does not mean that skills become irrelevant or that all aspects of the work become easier. Specialized expertise, teamwork, large infrastructures, complicated machinery, advanced technology, trouble-shooting, and organizational factors continue to be required when a design and engineering approach develops. Thus, even though the engineering approach of synthetic biology aims to make processes more systematic and more reproducible, this will not make it easier for anybody to engineer biology. Indeed, some aspects of the work may become more complex, and new skills may be required.

A useful analogy to aeronautical engineering was used at the workshop to illustrate this. Planes are built from a large number of well-characterized parts in a systematic way, but this does not mean that any member of the general public can build a plane, make it fly, and use it for commercial transportation. Thus, it is too simplistic to suggest that if synthetic biology becomes an engineering discipline it will necessarily become easier for anybody to engineer biological systems, including dangerous ones. More care needs to be taken in the interpretation of statements about how synthetic biology will lead to “de-skilling” and “make the engineering of biology easier.”

Furthermore, the experiences of iGEM teams tend to demonstrate the challenges of successfully performing synthetic biology experiments, and demonstrate the ongoing need for guided instruction and collective expertise. iGEM is the annual International Genetically Engineered Machine competition, which brings together undergraduate students from across a range of disciplines to work collaboratively to design and build biological systems and operate them in living cells. The iGEM competition is linked to the parts-based approach to synthetic biology through its contributions to the Registry of Standard Biological Parts, and provides a proof-of-principle for the synthetic biology agenda (21).

iGEM teams typically receive considerable guidance from senior faculty members and, while iGEM is a collaborative exercise, biologically trained students still tend to be the ones who have the central roles in daily laboratory activity. Balmer and Bulpin (22) describe the collaborative experiences of one undergraduate iGEM team:

Over the course of the project, as time pressures became more significant, it became natural, when assigning the activities of the day, for them to conduct the procedures in which they had each become experts, as otherwise it would require them teaching someone else. […] As one of them explained: “From the start I had the idea that I would take a main role in modelling but also get some experience in the lab. However, I quickly gave up on lab work after the first few weeks because *the time frame for the project we had was not enough to learn the basics needed for the lab and apply them*.” Owing to these contextual, material specificities of the laboratory and modelling work, *the sub-teams were quickly separated by knowledge, time and space* [(22), p. 14, emphasis added].

Cockerton (23) reported similar findings from her ethnography of two iGEM teams:

both teams found that many protocols were not streamlined as descriptions of synthetic biology often present. There was a great deal of tedious work, which involved small volumes of clear liquid and lots of waiting time. Many cycles of failed experiments had to be repeated (p. 306).

[…]

the reality of everyday design-experiment-fail-redesign (and so on…) cycles serves as a sobering reminder that the foundations of synthetic biology were not then (when I was in the field in 2009), and are not yet (2011), stable. Many experiments don’t work out as planned because many BioBricks from the Registry don’t function reliably. Presently, engineering that is accomplished with BioBricks in one lab and described in a standard fashion, certainly does not guarantee that the same result is reproducible in another lab (p. 307–308).

These in-depth analyses of synthetic biology in action illustrate the importance of collective expertise in synthetic biology research and the challenge posed by tacit knowledge, especially for wet lab work. Members of iGEM teams have or acquire distinct specialist sets of knowledge and skills, which are then applied to the collective project. Training by experienced professional researchers, and specialist skill sets acquired through trial and error, are still highly relevant to the success of synthetic biology projects.

The challenge of acquiring the specialist skill sets to perform laboratory work is also demonstrated by the experiences of some members of the DIY biology (DIYbio) community. DIYbio.org describes itself as “an organization dedicated to making biology an accessible pursuit for citizen scientists, amateur biologists, and biological engineers who value openness and safety”^3^. The organization comprises over 2000 members globally, although the actual number of members regularly conducting biological experimentation is much smaller. Some DIY biologists work in home laboratories assembled from everyday household tools and second-hand laboratory equipment purchased online. However, the majority conduct their experiments in community labs or “hackerspaces” (24).

DIY biologists typically comprise a wide range of participants of varying levels of expertise, ranging from complete novices with no prior background in biology, to trained scientists who conduct DIY experiments in their own time. The experiences of amateur DIY biologists demonstrate how a lack of indoctrination in the practices of biology can present significant challenges. As Revill and Jefferson (9) note:

For example, the London Biohacker group […] have noted the challenge of overcoming “pipetting errors” when trying to optimise techniques for DNA extraction and PCR process. MadLab, a bio group based at the Manchester Digital Laboratory, experienced similar difficulties during their “PCR challenge,” in which they pitched their home-made Arduino-based PCR machine against the open-source OpenPCR kit and the commercial PCR at Manchester Metropolitan University: …“the hardest part of the process was getting our samples into the gel using a micropipette. It turns out there is a bit of an art to pipetting … The more experienced pipettors claimed that it took them weeks to get the proper technique.” (p. 6)

Scientists typically build up these skills over the course of their training, but they present notable challenges for amateurs. Thus, while representations of Myth 1 imply that the material, informational aspects of synthetic biology will make it easier for anybody to exploit this technology to do harm, further examination of the social dimensions of scientific practice reveal the continued significance of local, specialized knowledge, and the importance of enculturation in laboratory practices.

At the workshop recently convened by the authors, an interesting tension was revealed. On the one hand, if tacit knowledge remains important in synthetic biology, then this implies that it will not be easily accessible to outsiders and this reduces concerns about the dual-use threat. On the other hand, if synthetic biology is an engineering discipline *and if*, as stated by Mukunda et al. in the citation above, this represents “an assault on the necessity of tacit knowledge” (17), then this implies that it will become more accessible to outsiders and this increases the dual-use threat. Thus, biosecurity concerns are heightened when more extreme depictions of synthetic biology’s ability to engineer biology are emphasized. We characterize this as the “synthetic biology/engineering conundrum” (11).

### Myth 2

#### Synthetic biology has led to the growth of a DIY biology community, which could offer dual-use knowledge, tools, and equipment for bioterrorists seeking to do harm

Developments in synthetic biology are seen to be closely associated with the growth of the DIYbio community, and concerns are expressed that this could offer knowledge, tools, and equipment to bioterrorists seeking to do harm. This was a key thrust in Carlson’s 2003 article, which started with the phrase: “The advent of the home molecular laboratory is not far off.” Schmidt also stressed this notion in his 2008 article, saying, for example: “[Imagine] a world where practically anybody with an average IQ would have the ability to create novel organisms in their home garage” [(16), p. 2]. This anticipated rise of a form of biology that could be performed by amateurs in their home garage or kitchen (25), sometimes referred to as “biohacking,” was understandably picked up by biosecurity experts. Jonathan Tucker, a well-recognized expert on chemical and biological weapons, wrote several articles on this topic, and in the most widely cited of these (cited 96 times according to Google Scholar, accessed 07/07/2014), he said:

The reagents and tools used in synthetic biology will eventually be converted into commercial kits, making it easier for biohackers to acquire them. Moreover, as synthetic biology training becomes increasingly available to students at the college and possibly high-school levels, a “hacker culture” may emerge, increasing the risk of reckless or malevolent experimentation [(26), p. 42].

Such concerns became prevalent at the NSABB, an organization established in 2005 to provide advice to the US government on biosecurity issues:

As synthetic biology techniques become easier and less expensive and the applications become more widely relevant, the range of practitioners expands to include scientists from a variety of disciplines; students at all levels, including high school; and amateur scientists and hobbyists who may lack any formal affiliations with universities or research institutions. The diversity of practitioners will also include individuals of different ages and varied social and educational backgrounds who may not have been sensitized to the ethical social and legal norms of the traditional life science research communities [(27), p. 11].

By 2014, this idea had become so widely accepted among experts in the field of Chemical, Biological, Radiological, and Nuclear (CBRN) weapons that an article entitled “DIY Bioterrorism Part II: the proliferation of bioterrorism through synthetic biology” was posted on the CBRNePortal.com. This article stated that:

The threat may be changing with the continued advancement of synthetic biology applications. Coupled with the ease of information sharing and a rapidly growing do-it-yourself-biology (DIYbio) movement, the chances of not only more attacks but potentially more deadly ones will inevitably increase (28).

#### Challenges to Myth 2

The link between synthetic biology and DIYbio, and the level of sophistication of the experiments typically being performed in DIYbio community labs, is overstated (24, 29). Members of DIYbio communities who are involved in more sophisticated experiments tend to be trained biologists, not amateurs and, as noted in the previous section, the experiences of amateur members of the DIYbio community demonstrate the challenges posed by tacit knowledge to successfully conduct even rudimentary biological experiments.

Furthermore, members of the DIYbio community tend to be proactive in addressing and engaging with safety and security concerns and many community labs have strict rules about access (24). For example, BioCurious, a community lab in silicon Valley, requires all members working in the wet lab to undertake a safety orientation, regardless of formal education or previous laboratory experience. BioCurious also has a safety committee that reviews requests to work with organisms not already on an approved list, and can approve, modify, or reject experimental design^4^.

DIYbio.org has also been active in promoting responsibility within the community. For example, in partnership with the Synthetic Biology Project at the Wilson Center, DIYbio.org has developed a Draft Code of Ethics that includes a focus on transparency, safety, and peaceful purpose^5^. In January 2013, DIYbio.org also launched an “Ask a Biosafety Officer” web portal^6^ in which anyone with a question can submit their query to a panel of volunteer biosafety experts. DIYbio Europe has established a set of Community Lab Safety Guidelines, with an emphasis on communication, openness, lab organization, and user and environmental safety (30). The US Federal Bureau of Investigation (FBI) weapons of mass destruction outreach program has also launched a series of efforts to promote outreach and oversight of the DIYbio community (31).

### Myth 3

#### DNA synthesis has become cheaper and can be out-sourced, and this will make it easier for terrorists to create biological threat agents

DNA synthesis is one of the key enabling technologies of synthetic biology. There are now a number of commercial companies that provide DNA synthesis services, so the process can be out-sourced: a client can order a DNA sequence online and receive the synthesized DNA material by post within days or weeks. The price charged by these companies has greatly reduced over the last 20 years, and is now around 0.3 US$ per base pair, which puts it within reach of a broad range of actors. This has led to routine statements suggesting that it is now cheap and easy to obtain a synthesized version of any desired DNA sequence. This popularized image of DNA synthesis is well represented by the Wikipedia entry (accessed 02/07/2014) for “artificial gene synthesis,” which states that: “it is possible to make a completely synthetic double-stranded DNA molecule with no apparent limits on either nucleotide sequence or size.”

Rob Carlson first published his now famous “Carlson curves,” illustrating the increasing productivity and reducing cost of DNA synthesis, in an article in the journal *Biosecurity and Bioterrorism*, which focused on how to combat the “potential for mischief or mistake” associated with advances in biological technologies (13). This illustrates how synthetic biology was, early on, promoted alongside discussions of a related biosecurity threat.

The key concern raised has been that bioterrorists could create dangerous viruses or other pathogens “from scratch,” meaning without access to the biological material from nature, from a strain repository, or from a laboratory. Instead, they would start with DNA or RNA genomic sequences for pathogenic viruses and bacterial pathogens that are increasingly freely available online. Such fears were heightened in 2002 by an experiment in which poliovirus was synthesized without the use of any natural virus or viral components (32). The research team, led by Eckard Wimmer, obtained published poliovirus RNA genome sequence information and converted this into DNA sequence data, which they then ordered from a commercial DNA synthesis company and assembled into a viral genome. The DNA was converted back into RNA and the RNA was used to produce a functional virus. Publication of this research in a scientific journal article immediately raised concerns that terrorists could use it as a recipe to synthesize dangerous viruses without needing access to biological material. These fears were further fueled when a journalist from *The Guardian* reported that he had been able to order online a synthesized DNA fragment from the smallpox virus genome and have it delivered to a residential address. According to this journalist, this showed “the ease with which terrorist organizations could obtain the basic ingredients of biological weapons” (33).

As Garfinkel et al. [(15), pp. 5–6) point out, although these experiments built upon previous work on DNA synthesis, “Wimmer’s work demonstrated for the first time in a post-September 11 world the feasibility of synthesizing a complete microorganism, in this case, a human pathogen – using only published DNA sequence information and mail-ordered raw materials.” Such concerns were further crystallized when, the following year, researchers at the JCVI similarly synthesized the bacteriophage phiX174 (a virus that infects bacteria) (34), and when researchers at the US Centers for Disease Control and Prevention “reconstructed” the Spanish flu virus (35), thought to have killed around 50 million people during the 1918 pandemic (36). This demonstrated that even viruses that could not otherwise be easily obtained in nature or from laboratory collections could be recreated (by well-resourced university researchers).

Together, the reconstruction of poliovirus and Spanish influenza virus have come to epitomize the threat narrative that DNA synthesis has become faster and cheaper, and that this will make it easier for terrorists to create biological threat agents. This is illustrated by statements from biosecurity experts such as Jonathan Tucker and Raymond Zilinskas^7^:

One potential misuse of synthetic biology would be to recreate known pathogens (such as the Ebola virus) in the laboratory as a means of circumventing the legal and physical controls on access to “select agents” that pose a bioterrorism risk. Indeed, the feasibility of assembling an entire, infectious viral genome from a set of synthetic oligonucleotides has already been demonstrated for poliovirus and the Spanish influenza virus [(26), p. 37].

Another article published in 2007 by Stephen Maurer and Laurie Zoloth stated that^8^:

Synthetic biologists have already shown how terrorists could obtain life forms that now exist only in carefully guarded facilities, such as polio and 1918 influenza samples [(37), p. 16].

In an early article highlighting this concern, security analysts from the Johns Hopkins Center for Civilian Biodefense Strategies wrote:

An editorial in a prestigious scientific journal reporting on the successful decoding and manipulation of the genetic sequence of the influenza A virus noted that “one can only speculate as to how quickly our knowledge….will progress, now that every nucleotide of the viral genome can be mutated and engineered back into the genome, in nearly endless combinations with other mutations.” […] Using such technologies, which have been utilized to investigate Ebola, pandemic flu, influenza, hanta viruses, lassa, rabies, and Marburg viruses, there is no need for a bioweaponeer to isolate the virus from an infected patient, acquire it from a germ bank, or culture it from nature. All the required starting materials, such as cell lines and DNA synthesizers, are widely available and used for many beneficent purposes. And the sequences for a growing variety of viruses that infect humans, animals and plants, including Ebola, pandemic influenza, and smallpox, are published in the open literature [(38), p. 30].

Tara O’Toole, Director of the Johns Hopkins Center for Civilian Biodefense Strategies and co-author of the article, was also the principal author of “Operation Dark Winter” (in 2001-2002) and “Atlantic Storm” (2005), the disaster response exercises that simulated covert outbreaks of smallpox in the United States. She went on to become Under Secretary of the Science and Technology Directorate of the Department of Homeland Security and, on the 10-year anniversary of the “anthrax letters,” reiterated her Johns Hopkins group’s earlier concerns with synthetic biology in testimony to the Senate Committee on Homeland Security and Governmental Affairs:

More than a decade ago, the Defense Science Board affirmed that, “there are no technical barriers to a large-scale bioattack.” We are living in the midst of a biotechnology revolution where the knowledge and tools needed to acquire and disseminate a biological weapon are increasingly accessible. It is possible today to manipulate pathogens’ characteristics (e.g., virulence, antibiotic resistance), and even to synthesize viruses from scratch. These procedures will inexorably become simpler and more available across the globe as technology continues to mature (39).

Concerns about terrorist use of DNA synthesis to create biological weapons spread internationally, and synthetic biology has become a regular feature of the science and technology reviews of the international treaty banning biological weapons: the BWC. In one of these reviews for BWC members, the Chinese delegation noted that:

With the spread of synthetic biology, some small scale research groups and even some individuals are now able to make the deadly Ebola and smallpox viruses and even some viruses against which all drugs are ineffective, thus making it much harder to counter bioterrorism. Furthermore, it has become much easier to obtain sensitive information. Using publicly available DNA sequences, terrorists can quickly synthesize pathogenic microbes that had previously been eradicated. [(40), p. 4].

During a 2012 Meeting of Experts of the BWC, the US delegation noted that:

These technologies [enabling technologies, including high-throughput systems for sequencing, synthesizing and analyzing DNA; bioinformatics and computational tools; and systems biology] could potentially be used for purposes contrary to the Convention, including making pathogens or toxins easier and less expensive to manufacture *de novo*, and further into the future, enabling development of biological weapons agents designed to evade countermeasures or target certain human populations [(41), p. 1-2].

Similar concerns have also been highlighted by individual bioweapons experts. Recent examples include Laurie Garrett’s^9^ article in the November/December 2013 issue of Foreign Affairs (42), which was widely disseminated and became the subject of a “Foreign Affairs Focus” video interview with the author published online on 15th January 2014^10^. In this article Garrett asserts that:

All the key barriers to the artificial synthesis of viruses and bacteria have been overcome, at least on a proof-of-principle basis (42).

Another example is the article written by Adam Bernier and Patrick Rose for the CBRNePortal, which states:

Non-state actors who wish to employ biological agents for ill intent are sure to be aware of how tangible bio-weapons are becoming as applications of synthetic biology become more affordable and the probability of success increases with each scientific breakthrough (28).

Synthetic biologists have not sought to deny these risks, and have led several initiatives to consider how these potential biosecurity risks could best be addressed. These initiatives re-enforced the association between synthetic biology, DNA synthesis, and biosecurity threats. For example in his “Synthetic Biohazard Non-proliferation Proposal,” George Church stated:

While the likelihood of misuse of oligos to gain access to nearly extinct human viruses (e.g. polio) or novel pathogens (like IL-4-poxvirus) is small, the consequences loom larger than chemical and nuclear weapons, since biohazards are inexpensive, can spread rapidly world-wide and evolve on their own (14).

Similarly, the JCVI report mentioned above concluded that:

today, any synthesis of viruses, even very small or relatively simple viruses, remains relatively difficult. In the near future, however, the risk of nefarious use will rise because of the increasing speed and capability of the technology and its widening accessibility. […] Ten years from now, it may be easier to synthesize almost any pathogenic virus than to obtain it through other means [(15), p. 12–13].

And a group of synthetic biologists (including Drew Endy and George Church) published, together with leading DNA synthesis companies and four FBI staff, a commentary in *Nature Biotechnology* on “DNA synthesis and biological security,” which stated that:

Like any powerful technology, DNA synthesis has the potential to be purposefully misapplied. Misuse of DNA-synthesis technology could give rise to both known and unforeseeable threats to our biological safety and security [(43), p. 627].

#### Challenges to Myth 3

When speaking about DNA synthesis, it is useful to distinguish between (a) the synthesis of oligonucleotides, commonly referred to as “oligos,” which are typically less than 100 nucleotides in length; (b) “gene synthesis,” a term used to refer to the *de novo* synthesis of “gene-length” DNA sequences, typically 200–3,000 base pairs (bp); and (c) the assembly of *de novo* synthesized gene-length fragments into genetic circuits and whole genomes.

There are a number of ways in which DNA synthesis could be used to create a synthetic viral genome [(44), p. 134]. An entire viral genome could be ordered online from a commercial gene synthesis company. Short, single stranded oligonucleotides could also be ordered from different gene synthesis companies and “stitched” together to create a complete viral genome. Alternatively, oligonucleotides could be synthesized using a purchased or custom-built DNA synthesizer, and these fragments could then be assembled into a complete viral genome. Several challenges should be taken into account when assessing the potential for this technology to be misused.

Ordering short oligos and then assembling them into a genome was the method used in the polio and Spanish flu experiments, but this required specialist expertise, experience, and equipment, which were all available in the academic laboratories involved but would not be easily accessible to an amateur working from home. Obtaining the oligos (as was done by *The Guardian* journalist for the smallpox virus) is only the first step in a complicated process. This is the first challenge to Myth 3.

The second challenge to Myth 3 is that, contrary to what is stated in Wikipedia, and what is often implied in the policy discourse described above, even specialized DNA synthesis companies cannot easily synthesize *de novo* any desired DNA sequence. Several commercial companies provide routine gene synthesis services for sequences under 3,000 bp, but length is a crucial factor, the process is error prone, and some sequences are recalcitrant to chemical synthesis (those that are “complex,” have high GC content, or result in the expression of particular proteins when cloned). Thus, in a recent review of large-scale *de novo* DNA synthesis, Kosuri and Church conclude that:

Today, reconstructions of complete viral and bacterial genomes are testaments of how far our synthetic capabilities have come. Despite the improvements, our ability to read DNA is better than our ability to write it [(45), p. 499].

The polio and phi174 viruses both have relatively small genomes, but these are still 7,400 and 5,400 bp, respectively. Thus, several *de novo* synthesized DNA fragments would have to be assembled in order to produce a full genome and (even if this was not already regulated by voluntary guidelines adopted by DNA synthesis companies) it would not be possible to simply order the full-length genome sequence of a small virus online.

The third challenge is that for sequences longer than 5–10 kb, *assembly* of DNA fragments becomes the crucial step, not *de novo* DNA synthesis. This was the major technological feat in the work conducted at the JCVI that produced the “synthetic” bacterial genome, and the “Gibson Assembly method” developed for that project is now widely used. The description of that work, however, demonstrates how the assembly of smaller fragments into larger ones and eventually into a functioning genome required substantial levels of expertise and resources, including those needed to conduct trouble-shooting experiments to identify and correct errors when assembled DNA constructs did not perform as expected (46).

The fourth challenge to Myth 3 relates to cost. The price of gene synthesis has declined greatly over the last 20 years, and the policy discourse that underlies biosecurity fears often implies that it will naturally become even cheaper over time, and thus widely affordable. The decline in price has, however, more or less stagnated around 0.3 US$ per base pair since 2008; and Carlson (47), Kosuri and Church (45), and Shetty (48) each discuss reasons why investment in this area may not be sufficient or well directed enough to generate further significant advances.

The fifth and fundamental challenge to Myth 3 is that constructing a genome size DNA fragment is not the same as creating a functional genome. In particular, ensuring the desired expression of viral proteins is a complex challenge, which has been well documented in Vogel’s (5) account of the 2002 poliovirus synthesis experiment. Drawing on interviews with the researchers involved in the experiment, Vogel found that making HeLa cell-free extracts was a crucial step in translating the synthetic genome into infectious virus particles; and it was also one of the most difficult parts of the experiment. Successful preparation of the HeLa cell-free extracts depended on craft-like techniques that require specialized and localized know-how. Yet, as Vogel notes, despite the difficulties encountered in this step of the process, published protocols of the experiment give no indication of this contingency:

As this case study illustrates, successful replication of the published 2002 poliovirus experiment hinges not only on the availability of the genetic sequence of the virus, commercial pieces of DNA, or the posting of the publication on the internet but also on the ability to master the mundane yet idiosyncratic biological techniques and adhere to specific laboratory disciplines [(5), p. 86].

Published accounts of science imply that experiments are readily replicable and transferrable from one lab to the next, but Vogel’s analysis demonstrates the significance of tacit knowledge in scientific practice and how this would limit the “proliferation” of skills anticipated in the dominant narrative on synthetic biology. Recognizing the importance of such tacit knowledge would enable more refined analyses of the potential biosecurity threat posed by advances in DNA synthesis technologies.

Additional challenges to Myth 3 include the fact that while DNA or RNA sequence data are available for many pathogenic viruses, genomes published in publicly available databases can contain errors or may be derived from attenuated laboratory strains (49). Producing viral particles in a laboratory is, moreover, not the same as creating and deploying an effective biological weapon. Challenges to the processes of scaling up, storage, and developing a suitable dissemination method are discussed under Myth 5.

### Myth 4

#### Synthetic biology could be used to design radically new pathogens

In addition to recreating dangerous viruses, concerns have also been expressed that synthetic biology could be used to enhance the virulence or increase the transmissibility of known pathogens in order to create novel threat agents.

The 2001 mousepox experiment is the most widely cited examples of the dual-use potential of life science research and has come to epitomize the potential to create more virulent viruses. In this experiment, researchers inserted the gene for interleukin-4 into the mousepox virus (50). They aimed to produce an altered virus that would induce infertility in mice and serve as an infectious contraceptive for pest control. However, the altered virus was found to be lethal to mice. Moreover, and most surprisingly, it was lethal to mice that were naturally resistant to mousepox as well as to mice that had been recently immunized against ordinary mousepox. The publication of these findings led to concerns that they could provide instructions to terrorists to produce novel biological weapons.

An early, formative report that shaped concerns about radically new pathogens was *Biotechnology Research in an Age of Terrorism* from the US National Research Council. It noted:

The effects of naturally occurring pathogens are limited by the evolutionary advantage gained by not eliminating their hosts. Among the many implications of the anticipated progress in biotechnology is the presumption that it may be feasible to create novel biological agents that are far more predictable and dangerous than any of the naturally occurring pathogens that have been developed as biological weapons in the past. It may be difficult to engineer a more successful pathogen than those already present in nature that have been perfected by evolution for their niche in life. However, application of the new genetic technologies makes the creation of “designer diseases” and pathogens with increased military utility more likely [(51), p. 25].

These concerns have been echoed in a number of other high profile reports. For example, the very first European Commission report dedicated to synthetic biology, published in 2007, stated that:

The possibility of designing a new virus or bacterium “*à la carte*” could be used by bioterrorists to create new resistant pathogenic strains or organisms, perhaps even engineered to attack genetically specific sub-populations [(52), p. 18].

A 2012 report from United Nations Interregional Crime and Justice Research Institute (UNICRI) raised similar concerns:

Experts felt that as an enabling tool, synthetic biology […] would in the long term likely facilitate the work of those attempting to acquire and use biological weapons. More dangerous and controllable pathogens could be engineered that lead to novel possibilities in designing bioweapons. Advances in modeling could enable improvements in weapons design. Metabolic engineering might confer new qualities and attributes upon agents and offer options for new types of weapons. […] This could have the negative effect of making bioweapons cheaper and easier to acquire, making their use eventually more likely; more reliable and controllable, making them more desirable; and more effective, increasing their potential impact [(53), p. 34].

These concerns are also evident in the statements made by the Chinese and US delegations in the BWC reports identified under Myth 3.

Influential experts have also highlighted concerns about “super-pathogens,” for instance Marc Collett, a virologist who was commissioned by the JCVI to provide advice for their work on the risks and benefits of synthetic genomics, concluded that:

While nature has provided would-be terrorists an ample supply and selection of quite virulent viruses, there is concern that genetic technologies will be used to modify these already pathogenic agents and create “super-pathogens,” viruses that are more lethal and disruptive than naturally occurring pathogens, and that are designed to evade vaccines or to be resistant to drugs [(54), p. 95].

Maurer and Zoloth, in the article mentioned above, similarly stated that:

Synthetic biology’s efforts to reprogram life have raised concerns in some quarters that the technology could one day be used to make radically new weapons, such as pathogens that could be narrowly targeted towards populations with known genetic susceptibilities [(37), p. 16].

Laurie Garrett, in her 2013 article for *Foreign Affairs*, raised her concerns as follows:

a simple, ubiquitous microbe such as *E. coli*, a bacterium that resides in the guts of every human being, can now be transformed into a killer germ capable of wreaking far more havoc than anything on [the US National Select Agent] registry (42).

The 2011–2012 controversy over publication of H5N1 “bird-flu” research also centered on concerns that the published research would provide “blueprints” to terrorists to create highly virulent viruses with increased transmissibility. H5N1 does not spread easily from human to human, but it kills between 30 and 80% of people infected (55). In this experiment, researchers in the Netherlands and the US independently developed a novel strain of the H5N1 avian influenza virus that could spread more easily to humans and other mammals. They passed H5N1 among ferrets and found that a mutated H5N1 virus that was air transmissible could emerge, and that this variant was still highly virulent. When two papers relating similar experimental results were submitted for publication to *Science* and *Nature*, concerns were raised about the dual-use risk and the NSABB recommended against full publication of the study. After additional consultations at the World Health Organization, the NSABB reversed its position and recommended publication of revised versions of the papers (56).

#### Challenges to Myth 4

The mousepox and H5N1 experiments are frequently cited to demonstrate how dangerous new pathogens could be designed. However, assessments of this threat tend to overlook the fact that, in both these experiments, the researchers did not actually *design* the pathogens. With respect to H5N1, researchers had indeed been trying to design an air-transmissible virus variant for some time, without success. The ferret experiment was set up as an alternative approach, to see whether “natural” mutations could generate an air-transmissible variant. The researchers had no influence on the specific mutations induced. In the IL4 mousepox experiment, the results were unanticipated by the researchers. In other words, they were not planned for.

Moreover, some of the key lessons that came out of the extensive Soviet program to weaponize biological agents were about the trade-offs between improving characteristics that are “desired,” in the context of a bioweapons program, such as virulence, and diminishing other equally “desired” characteristics, like transmissibility or stability. One project, for example, aimed to develop strains of *F. tularensis* (which causes tularemia) that were resistant to current vaccines and to multiple antibiotics. Genes coding for antibiotic resistance were successfully transferred into *F. tularensis*, but the new strain lost its virulence. Domaradsky, who led the research, wrote:

Everyone who has ever dealt with the genetics of bacteria knows how complicated it is to produce a new strain, indeed, to create a new species! [quoted in (57), p. 186)].

The Soviets did, however, eventually succeed in developing a strain of *F. tularensis* that was resistant to multiple antibiotics and retained its pathogenic characteristics. They also worked on four additional bacterial strains – *B. anthracis* (which causes anthrax), *B. mallei* (glanders)*, B. pseudomallei* (melioidosis), and *Y. pestis* (plague) – with the goal of making each of them resistant to 10 antibiotics, but this proved too technically difficult. As Leitenberg and Zilinskas note in their account of the process:

The most difficult problems had to do with pleiotropic effects and a lack of stability in engineered strains. Antibiotic-resistant cells had a distressing habit of losing virulence or exhibiting lesser yields (or both) when propagated in culture. As for stability […] when the construct for resistance to one antibiotic was introduced into the host cell, an earlier emplaced construct was often lost. This sort of problem required additional rounds of research, which were both labor intensive and time consuming [(57), p. 188].

Pleiotropic effects (where a single gene affects more than one characteristic) and genetic instability are common in microorganisms, and while it is too simple to say that increased transmissibility will always be associated with reduced virulence, this is often the case for strains produced in laboratories. In the case of viruses, this is in part because the production of virus molecules necessitates passage through a series of host organisms, and that during this scaling-up process the virus is not subject to any evolutionary pressure to maintain virulence, and thus – although this cannot be taken as a definitive rule – the virus tends to accumulate mutations that generate an attenuated strain. Similarly, bacteria cultured in laboratories will tend to lose virulence.

### Myth 5

#### Terrorists want to pursue biological weapons for high consequence, mass casualty attacks

Underlying the first four myths are certain assumptions about who the terrorists might actually be, what their intentions are, what capabilities they might pursue, and the level of skills and resources available to them. Despite a lack of analysis of the potential adversaries involved in the misuse of life science research, the bioterrorism threat has generally been portrayed in policy circles as an imminent concern, and emphasis is placed on high consequence, mass casualty attacks, performed with “weapons of mass destruction” (WMD).

For example, in one of the President George W. Bush’s earliest statements following 9/11 and the “anthrax letter” attacks that drew the American people’s attention to the biological weapons threat, he said:

Since September 11, America and others have been confronted by the evils these [biological] weapons can inflict. This threat is real and extremely dangerous. Rogue states and terrorists possess these weapons and are willing to use them (58).

Later, he set up a WMD Commission and tasked it with examining the threat posed by the nexus of international terrorism and the proliferation of weapons of mass destruction. In its report, this Commission asserted:

Unless the world community acts decisively and with great urgency, it is more likely than not that a weapon of mass destruction will be used in a terrorist attack somewhere in the world by the end of 2013. The Commission further believes that terrorists are more likely to be able to obtain and use a biological weapon than a nuclear weapon. The Commission believes that the U.S. government needs to move more aggressively to limit the proliferation of biological weapons and reduce the prospect of a bioterror attack [(59), p. xv].

Bioterrorism became one of the Bush Administration’s key security concerns over its two terms in office. One estimate of civilian biodefense expenditure across the federal government since 2001 is that more than $70 billion have been spent (60). Despite this, on the 10-year anniversary of 9/11 and the “anthrax letter” attacks, the former US senators who chaired the WMD Commission, Bob Graham and Jim Talent, released a “report card” on America’s bio-response capabilities that concluded the US was still unprepared to respond to large-scale biological attacks. It also warned:

Naturally occurring disease remains a serious biological threat; however, a thinking enemy armed with these same pathogens — or with multi-drug-resistant or synthetically engineered pathogens — could produce catastrophic consequences. A small team of individuals with graduate training in several key disciplines, using equipment readily available for purchase on the Internet could produce the type of bioweapons created by nation-states in the 1960s. Even more troubling, the rapid advances in biotechnology, such as synthetic biology, will allow non-state actors to produce increasingly powerful bioweapons in the future [(61), p. 11].

We see here how the myths we previously discussed, about de-skilling and increased access, and about the ease of designing new dangerous pathogens, underlie concerns about terrorists’ potential ability to launch a mass attack, and how these are connected, by actors, with the advent of synthetic biology.

The senators were not alone in their assessments. For instance, the US Senate Majority Leader Bill Frist made a similar warning in an earlier speech outlining the global threat of infectious disease and bioterrorism, and the need to better prepare the US and the world to respond to epidemics and outbreaks:

No intelligence agency, no matter how astute, and no military, no matter how powerful and dedicated, can assure that a few technicians of middling skill using a few thousand dollars worth of readily available equipment in a small and apparently innocuous setting cannot mount a first-order biological attack … Never have we had to fight such a battle, to protect so many people against so many threats that are so silent and so lethal (62).

Similar messages were reinforced at the highest level. Addressing BWC members at their five-yearly meeting in 2011, Secretary of State Hillary Clinton said:

The advances in science and technology make it […] easier for states and non-state actors to develop biological weapons. A crude, but effective, terrorist weapon can be made by using a small sample of any number of widely available pathogens, inexpensive equipment, and college-level chemistry and biology (63).

She also acknowledged, however, that not everyone in the international community shared the US assessment:

I know there are some in the international community who have their doubts about the odds of a mass biological attack or major outbreak. They point out that we have not seen either so far, and conclude the risk must be low. But that is not the conclusion of the United States, because there are warning signs, and they are too serious to ignore (63).

The belief that the focus should be on mass attacks was bluntly stated by an FBI agent at a symposium on synthetic biology this year (1st May), when she warned: “These technologies do not just pose a risk to individual buildings or cities, but if cleverly deployed, can reduce our population by significant percentages” (64).

#### Challenges to Myth 5

There are two dimensions to Myth 5. The first is about the intention of would-be terrorists, and the assumption is that terrorists would seek to produce mass casualty weapons and pursue capabilities on the scale of twentieth century state-level bioweapons programs. While most leading biological disarmament and non-proliferation experts believe that the risk of a small-scale bioterrorism attack is very real and very present, they consider the risk of sophisticated large-scale bioterrorism attacks to be very small (65). This is backed up by historical evidence. The three confirmed attempts to use biological agents against humans in terrorist attacks in the past were small-scale, low casualty events aimed at causing panic, and disruption rather than excessive death tolls: (i) the Rajneesh cult’s use of *Salmonella* on salad bars in local restaurants to sicken potential voters and make them stay away from the polls during Oregon elections in 1984; (ii) the 1990–95 attempted use of botulinum toxin and anthrax by the Japanese Aum Shinrikyo cult; (iii) and the “anthrax letters” sent to media outlets and members of US Congress in 2001 resulting in at least 22 cases of anthrax, five of which were fatal (66, 67).

The second dimension to Myth 5 is the implicit assumption that producing a pathogenic organism equates producing a weapon of mass destruction. It does not. Considerable knowledge and resources are necessary for the processes of scaling up, storage, and developing a suitable dissemination method. These processes present significant technical and logistical barriers. Drawing from her in-depth study of the Iraqi, Soviet, and US bioweapons programs (3, 4), Ben Ouagrham-Gormley explains:

Scaling up fragile microorganisms that are sensitive to environmental conditions and susceptible to change — and viruses are more sensitive than bacteria — has been one of the stiffest challenges for past bioweapons programs to overcome, even with appropriate expertise at hand. Scaling-up requires a gradual approach, moving from laboratory sample, to a larger laboratory quantity, to pilot-scale production, and then to even larger-scale production. During each stage, the production parameters need to be tested and often modified to maintain the lethal qualities of the agent; the entire scaling-up process can take several years (68).

The dissemination of biological agents also poses difficult technical challenges. Whereas persistent chemical agents such as sulfur mustard and VX nerve gas are readily absorbed through the intact skin, no bacteria and viruses can enter the body via that route unless the skin has already been broken. Biological agents must either be ingested or inhaled to cause infection. To expose large numbers of people through the gastrointestinal tract, possible means of delivery are contamination of food and drinking water, yet neither of these scenarios would be easy to accomplish. Large urban reservoirs are usually unguarded, but unless terrorists added massive quantities of biological agent, the dilution effect would be so great that no healthy person drinking the water would receive an infectious dose (66). Moreover, modern sanitary techniques such as chlorination and filtration are designed to kill pathogens from natural sources and would probably be equally effective against a deliberately released agent. Bacterial contamination of the food supply is also unlikely to inflict mass casualties. Cooking, boiling, pasteurization, and other routine safety precautions are generally sufficient to kill pathogenic bacteria.

The most likely way to inflict mass casualties with a biological agent is by disseminating it as a respirable aerosol: an invisible cloud of infectious droplets or particles so tiny that they remain suspended in the air for long periods and can be inhaled by large numbers of people. A high-concentration aerosol of *B. anthracis* or some other pathogen, released into the air in a densely populated urban area, could potentially infect thousands of victims simultaneously. After an incubation period of a few days, depending on the type of agent and the inhaled dose, the exposed population would experience an outbreak of an incapacitating or fatal illness. Although aerosol delivery is potentially the most lethal way of delivering a biological attack, it involves major technical hurdles that most terrorists would be unlikely to overcome. To infect through the lungs, infectious particles must be microscopic in size – between 1 and 5 μm in diameter. Terrorists would therefore have to develop or acquire a sophisticated delivery system capable of generating an aerosol cloud with the necessary particle size range and a high enough agent concentration to cover a broad area. Overall, an important trade-off exists between ease of production and effectiveness of dissemination. The easiest way to produce microbial agents is in a liquid form, yet when such a “slurry” is sprayed into the air, it forms heavy droplets that fall to the ground so that only a small percentage of the agent is aerosolized. In contrast, if the bacteria are first dried to a solid cake and then milled into a fine powder, they become far easier to aerosolize, yet the drying and milling process is technically difficult.

The Aum Shinrikyo cult struggled with dissemination (67, 69, 70). In one of its anthrax dissemination attempts, it sprayed unknown, but probably very large, quantities of a liquid aerosol (most likely crude culture, unprocessed in any way) of *B. anthracis* from the roof of the Aum’s headquarters building in Tokyo. For the dissemination, the Aum set up two sprayers on the roof of the eight-story building, each within a large round cooling tower. Pipes were extended from the cooling towers to tanks below, which were filled with a liquid suspension of *B. anthracis*. The device worked poorly, producing large droplets rather than the very fine aerosol needed for effective transmission of anthrax. It also appears the spore concentration was very low (at least five orders of magnitude below that necessary for a highly infectious wet aerosol).

In another dissemination attempt, targeting the area around the Kanagawa prefectural office and the Imperial Palace, the Aum equipped vehicles with spraying devices, but according to prosecutors’ statements, the nozzle of the sprayer clogged and the operation failed. Despite its 200 m^2^ laboratory containing, amongst other equipment, a glove box, incubator, centrifuge, drier, DNA/RNA synthesizer, electron microscope, two fermenters each having about a 2,000 litre capacity, and an extensive scientific library, and despite its repeated attempts at dissemination, the Aum was unsuccessful in causing any disease, and in retrospect it is clear that the cult did not even make the first substantive step toward an effective bioweapon.

If, despite the odds, aerosolization was achieved, the effective delivery of biological agents in the open air is highly dependent on atmospheric and wind conditions, creating additional uncertainties. Only under highly stable atmospheric conditions would the aerosol cloud remain close to the ground where it can be inhaled, rather than being rapidly dispersed. Moreover, most microorganisms are sensitive to ultraviolet radiation and cannot survive more than 30 min in bright sunlight, limiting their use to night-time attacks. One major exception is anthrax, which can be induced to form spores with tough outer coats that enable them to survive for several hours in sunlight. Terrorists could, of course, stage a biological attack inside an enclosed space such as a building, a subway station, a shopping mall, or a sports arena. Such an attack, if it involved a respiratory aerosol, might infect thousands of people, but even here the technical hurdles would by no means be trivial.

Finally, even if a biological weapon had been disseminated successfully, the outcome of an attack would be affected by factors like the health of the people who are exposed to the agent, and the speed and manner with which public health authorities and medical professionals detected and were able to respond to the resulting outbreak. A prompt response with effective medical countermeasures, such as antibodies and vaccination, can significantly blunt the impact of an attack. Simple, proven ways to curtail epidemics, such as wearing face masks, hand washing, and avoiding hospitals where transmission rates might soar, can also prove effective in stemming the spread of a disease. Indeed, this aspect of a bioterrorism attack is often underplayed in scenarios like Tara O’Toole’s “Dark Winter” and “Atlantic Storm,” where the rates of contagion used are often significantly higher than those in historical cases of natural outbreaks (71).

## Discussion

We have identified a number of assumptions that underlie policy discourse on the biosecurity threat posed by synthetic biology. We characterize these assumptions as “myths” that pervade discussion on this issue and have identified important challenges to those myths. In particular, we argue that the myths overlook significant difficulties faced when seeking to design and/or produce a pathogen because they focus mostly on material features, thus missing important socio-technical factors, such as tacit knowledge. We have also shown that this dominant narrative underestimates a crucial step needed to mount a terrorist attack, especially a mass attack: the need to produce weapons, not just pathogens. Thus, we conclude that the five myths that recur in the dominant narrative embody misleading assumptions about both synthetic biology and bioterrorism.

The purpose of identifying and challenging these “myths” is not to dismiss the threat of a bioweapons attack. Of course, it is prudent to take measures to prepare against the possibility of a biological weapons attack and concerted action across a policy continuum that extends from prevention through preparedness to consequence management is necessary. However, as we have demonstrated, any bioterrorism attack will most likely be one using a pathogen strain with less than optimal characteristics disseminated through crude delivery methods under imperfect conditions, and the potential casualties of such an attack are likely to be much lower than the mass casualty scenarios frequently portrayed. This is not to say that speculative thinking should be discounted as it can, in some policy contexts, be helpful to represent possible, though not necessarily probable, future scientific developments, in order to encourage thinking on long-term security challenges. However, problems arise when these speculative scenarios for the future are distorted and portrayed as scientific reality in the present, which, as this paper demonstrates, has occurred in policy narratives related to synthetic biology and biosecurity.

We have shown that much of the debate in policy forums about the biosecurity threat of synthetic biology is based on naïve and simplistic interpretations of synthetic biology’s ability to “make biology easier to engineer,” and in particular on the misleading assumption that the skills and knowledge necessary to perform synthetic biology will necessarily become accessible to people with no specialist expertise working outside professional scientific institutions, including hostile actors who would seek to misuse the technology to develop biological weapons.

In order to understand why such myths develop and persist, it is important to consider the role that they play in the social dynamics of synthetic biology. Drawing on the literature in the sociology of expectations (72), we suggest that particular portrayals of synthetic biology are mobilized by various actors – deliberately or not – to strengthen their own perspectives and interests, and to help bring into being their own “hoped-for” future. The myths act as “prospecting retrospects”: prospects that are deployed in the real-time now, in order to construct particular futures (72). Discourses about the future are *performative*, meaning that they “perform” functions (they “do work”) and are also *relational*, meaning that they bind together and enroll actors and other resources into networks (73). Thus, discourse is “wishful enactment” not just “wishful thinking” (74).

With respect to synthetic biology, different communities of actors stress particular issues in particular contexts. This frames the debate in particular ways and plays an important role in constructing and maintaining resources and support for each of these communities. For example, scientists such as Rob Carlson, George Church, or Drew Endy, who are heavily engaged in the promotion of synthetic biology, need to portray an optimistic vision of the potential of the engineering approach to biology as part of their endeavors to develop support for a new field of research which they believe has great significance and potential. Actors in the security field (including some policy makers, social scientists, and natural scientists) play a different role and often exaggerate the “dual-use threat” in order to attract attention and resources to their own work. Researchers from our own field of science and technology studies (STS) are not immune from such processes: we will generally seek to emphasize the complexity of real world situations and the importance of social dimensions of science, in order to justify the need for our expertise. However, at least until now, STS framings have had less influence on the dominant narrative than the discourse mobilized by actors from the fields of synthetic biology and biosecurity. Thus, the myths we have discussed in this paper have played an important role in defining synthetic biology as a “promissory” field of research and as an “emerging science and technology” in need of scenario forecasting, regulation and governance. Our aim is not to denigrate the behavior of those who deploy these narratives. Rather, we suggest that when discourse is understood as something that seeks to change the social world, we can move beyond the battle that we have regularly encountered in discussions about synthetic biology, that focuses on whose prognosis is most accurate and whether or not “it is just hype” (19, 20).

We believe that a better understanding and acknowledgment of the social dynamics at play would help to develop more productive discussions in which the different communities involved could move beyond simply promoting their own interests and perspectives. This is important because in some cases the discourse deployed can have unintended consequences that are detrimental to the interests of the actors themselves, and to the nature of public debate. Thus, overstating the “promise” of synthetic biology applications manifestly leads to parallel overstatements about the “perils” of the field: the promissory discourse of synthetic biology is bolstered by the “promised peril” of misuse by malevolent actors. The fact that these myths (or at least the first 4) serve to bolster the positive promises of synthetic biology helps to explain why these myths continue to persist, despite the fact that they do not accurately reflect current or foreseeable realities for the practice of synthetic biology. This is somewhat incongruous since the hoped-for futures of the actors who promote the benevolent development of synthetic biology do not, of course, include large-scale fatal bioterrorist attacks.

If we are to disentangle synthetic biology and biosecurity concerns, and to have a more refined assessment of both the biosecurity threat and the anticipated benefits, we believe that it is necessary to have more nuanced discussions about the extent to which synthetic biology is, or ever will be, an engineering discipline, and whether, in practice, this would reduce the importance of tacit knowledge, specialist expertise of different kinds, collective work, large infrastructures, and organizational factors. Such discussions would need to identify those aspects of the work that would become easier – in the sense that they can, for example, be automated and reliably performed by a robot – and those which are likely to remain difficult, in the sense that they still require craft skills to be successfully achieved. This would need to take into account not only the material and informational aspects but also other important socio-technical dimensions that will shape the development of the field.

## Author Contributions

The three authors are listed in alphabetical order and have all contributed equally to this paper, including the conception, analysis and data collection of the research, drafting the text, and revising it critically for intellectual content.

## Conflict of Interest Statement

This research was conducted at the Centre for Synthetic Biology and Innovation (CSynBI), which is a partnership between synthetic biology researchers at Imperial College London and social scientists at the Department of Social Science, Health and Medicine at King’s College London. CSynBI seeks to influence the trajectory of synthetic biology and contribute to the development of an appropriate governance regime.

## Footnotes

^1^A list of some of our higher profile engagements can be found here: http://www.kcl.ac.uk/sspp/departments/sshm/research/Research-Labs/CSynBI@KCL-Impact.aspx.

^2^Gautam Mukunda is now at the Harvard Business School.

^3^http://diybio.org/about-2/, accessed 14/07/2014

^4^http://biocurious.org/faq/, accessed 14/07/2014

^5^http://diybio.org/codes, accessed 14/07/2014

^6^http://ask.diybio.org, accessed 14/07/2014

^7^Jonathan B. Tucker was at this time a senior fellow at the Center for Nonproliferation Studies (CNS) of the Monterey Institute of International Studies, where he specialized in biological and chemical weapons issues. Raymond Zilinskas was and still is the director of the Chemical and Biological Weapons Nonproliferation Program at CNS.

^8^Stephen Maurer was then and still is at the University of California-Berkeley’s Goldman School of Public Policy and Director of the Goldman School Project on Information Technology and Homeland Security. Laurie Zoloth was and still is Professor of Bioethics at Northwestern University.

^9^Laurie Garrett is a science writer with a special interest in emerging infectious diseases, global health and biosecurity. She works at the Council on Foreign Relations Council, a think-tank that publishes the journal *Foreign Affairs*.

^10^http://www.foreignaffairs.com/discussions/audio-video/foreign-affairs-focus-laurie-garrett-on-synthetic-biology
